# The Return of National Self-Sufficiency? Excavating Autarkic Thought in a De-Globalizing Era

**DOI:** 10.1093/isr/viaa092

**Published:** 2021-01-05

**Authors:** Eric Helleiner

**Affiliations:** Waterloo, Canada

**Keywords:** autarky, self-sufficiency, de-globalization, Palabras clave, autarquía, autosuficiencia, desglobalización, Mots clés, autarcie, autosuffisance, démondialisation

## Abstract

As the global crisis triggered by the COVID-19 virus unfolded, *The Economist* magazine published a cover in May 2020 titled “Goodbye globalization: the dangerous lure of self-sufficiency.” The title summed up well the new political interest in the ideology of national economic self-sufficiency in the pandemic context. Unfortunately, contemporary textbooks in the field of international political economy (IPE) say little about this kind of “autarkic” thought. No survey of the history of autarkic thought exists even within specialist IPE literature or in the fields of intellectual history and the history of economic thought. Filling this gap in existing scholarship, this article highlights a rich history of autarkic thought that includes the ideas of famous thinkers such as Jean-Jacques Rousseau, Johann Fichte, Mohandas Gandhi, and John Maynard Keynes. Three core rationales for a high degree of national self-sufficiency have been advanced in the past: (1) insulation from foreign economic influence, (2) insulation from foreign political and/or cultural influence, and (3) the promotion of international peace. At the same time, considerable disagreements have existed among autarkists about some of these rationales and their relative importance, as well as about the precise meaning of national self-sufficiency. These disagreements stemmed not just from differences in their specific goals but also from the different conditions across time and space in which autarkic thought was developed. In addition to improving understanding of the autarkic ideological tradition, this article contributes to emerging scholarship attempting to overcome Western-centrism in IPE scholarship as well as literature exploring the new politics of de-globalization in the current era.

## Abstract (es)

A medida que se desarrollaba la crisis mundial que desencadenó el virus causante de la COVID-19, en mayo de 2020, la revista The Economist publicó una portada con el título “Adiós a la globalización: el peligroso señuelo de la autosuficiencia.” El título es un buen resumen del nuevo interés político en la ideología de la autosuficiencia económica nacional en el contexto de la pandemia. Desafortunadamente, los libros de texto actuales del campo de la economía política internacional (EPI) dicen poco sobre este tipo de pensamiento “autárquico.” No existe ningún estudio de la historia del pensamiento autárquico, ni siquiera en la literatura especializada en EPI ni en los campos de la historia intelectual y la historia del pensamiento económico. Para llenar el vacío que existe en los estudios académicos, este artículo hace hincapié en la rica historia del pensamiento autárquico, que incluye las ideas de pensadores famosos como Jean-Jacques Rousseau, Johann Fichte, Mohandas Gandhi y John Maynard Keynes. En el pasado se han propuesto tres razones fundamentales para que exista un alto grado de autosuficiencia nacional: (1) el aislamiento de la influencia económica extranjera, (2) el aislamiento de la influencia política o cultural extranjera, y (3) la promoción de la paz internacional. Al mismo tiempo, entre los autarquistas han existido considerables desacuerdos sobre algunos de estos fundamentos y su importancia relativa, así como sobre el significado preciso de “autosuficiencia nacional.” Estos desacuerdos surgieron no solo de las diferencias entre sus objetivos específicos, sino también de las diferentes condiciones de tiempo y espacio en las que se desarrolló el pensamiento autárquico. Además de mejorar la comprensión de la tradición ideológica autárquica, este artículo contribuye a la investigación emergente que intenta superar el centrismo occidental en los estudios académicos sobre EPI, como también en la literatura que explora las nuevas políticas de desglobalización en la era actual.

## Abstract (fr)

En cette crise mondiale déclenchée par le COVID-19, en mai 2020, « Au revoir la mondialisation: le leurre dangereux de l'autosuffisance » figurait en couverture du magazine The Economist. Ce titre résumait bien le nouvel intérêt politique pour l'idéologie de l'autosuffisance économique nationale dans le contexte de la pandémie. Malheureusement, les ouvrages modernes dans le domaine de l’économie politique internationale n'abordent que très peu ce type de pensée « autarcique ». Aucune étude sur l'histoire de la pensée autarcique n'existe, y compris dans la littérature spécialisée portant sur l’économie politique internationale ou sur des domaines tels que l'histoire intellectuelle et l'histoire de la pensée économique. Cet article comble cette lacune des études existantes en mettant en évidence la richesse de l'histoire de la pensée autarcique, qui englobe les idées de penseurs célèbres tels que Jean-Jacques Rousseau, Johann Fichte, Mohandas Gandhi et John Maynard Keynes. Trois principales raisons pour un haut niveau d'autosuffisance nationale ont été avancées par le passé: (1) une isolement par rapport à l'influence économique étrangère, (2) un isolement par rapport à l'influence politique et/ou culturelle étrangère, et (3) la promotion de la paix internationale. Dans le même temps, des désaccords considérables ont existé entre autarcistes au sujet de certaines de ces raisons et de leur importance relative ainsi que de la signification précise d'Autosuffisance nationale. Ces désaccords provenaient non seulement de différences dans leurs objectifs spécifiques, mais aussi des différentes conditions spatiales et temporelles dans lesquelles la pensée autarcique s'est développée. En plus de permettre une meilleure compréhension de la tradition idéologique autarcique, cet article contribue aux études émergentes tentant de surmonter le centrisme occidental des recherches en économie politique internationale, ainsi qu’à la littérature explorant les nouvelles politiques de démondialisation de l’époque actuelle.

The global crisis triggered by the COVID-19 virus has generated new political interest across the world in the cultivation of greater national economic self-sufficiency. This goal had already begun to attract support in some quarters before the crisis, but the economic vulnerabilities exposed by border closings and nationalist policies during the pandemic have placed it center stage in public policy debates around the globe. Even avid supporters of economic globalization have been forced to recognize the new widespread support for controls on cross-border economic flows aimed at this objective. As *The Economist* (2020) put it in May 2020, “don't expect a quick return to a carefree world of unfettered movement and free trade. The pandemic will politicise travel and migration and entrench a bias towards self-reliance.”

If this “bias towards self-reliance” persists, students of international political economy (IPE) need to better understand this new ideological landscape. Unfortunately, however, contemporary IPE textbooks do not say much, if anything, about the ideology of national economic self-sufficiency. To be sure, many of them identify “economic nationalism” as a longstanding ideological tradition in the field alongside that of economic liberalism and Marxism. However, this label is identified with historical figures such as the nineteenth-century German thinker Friedrich List who advanced a “neomercantilist” version of economic nationalism rather than an autarkic one. Neomercantilists call for targeted trade restrictions and other forms of government economic activism in order to promote domestic industries that can compete successfully in world markets. Theirs is an outward-oriented form of economic nationalism that seeks to boost their country's wealth and power in the context of an open world economy. Autarkists, by contrast, are focused inward on the goal of minimizing international economic linkages.^1^

In the context of the acceleration of economic globalization in the last few decades, the absence of serious attention to autarkic thought in IPE textbooks was understandable. Why should students study a set of beliefs that appeared anachronistic and to have so little political influence outside of unusual places such as North Korea? In this new world of de-globalization, however, the neglect is no longer justified. But where can IPE instructors turn to find a history of thought about the idea of national self-sufficiency? Within specialist IPE literature itself, the ideas of a few specific autarkic thinkers have received some attention, but no survey of the intellectual history of this ideology exists. Strikingly, the same is even true of broader literature in the fields of intellectual history or the history of economic thought.

This article begins to fill this existing gap in scholarship. I show how there is, in fact, a rich intellectual history to be explored on this topic that includes the ideas of famous thinkers such as Jean-Jacques Rousseau, Johann Fichte, Mohandas Karamchand Gandhi, and John Maynard Keynes. These figures rarely called for complete economic autarky (in the same way that few economic liberals endorse completely free movement of labor, capital, goods, and services across borders). However, their ideas can be described as “autarkic” because they each highlighted the benefits of cultivating a high degree of national self-sufficiency as an ideal. I demonstrate how these thinkers, taken together, identified three core rationales for this policy: (1) insulation from foreign economic influence, (2) insulation from foreign political and/or cultural influence, and (3) the promotion of international peace. At the same time, I demonstrate how autarkists disagreed about some of these rationales and their relative importance, as well as about the precise meaning of national self-sufficiency. These disagreements stemmed not just from differences in their specific goals but also from the different conditions across time and space in which autarkic thought was developed.

In addition to improving understanding of the history of autarkic thought, the article also seeks to contribute to two other emerging bodies of IPE literature. The first is scholarship attempting to challenge and transcend Western-centrism in the field (e.g., Phillips 2005; Leander 2015; Tussie and Riggiorzzi 2015; Deciancio and Quilconi 2020). I show how this goal is particularly important for any analysis of the history of autarkic thought because key contributions to this intellectual tradition came from non-Western thinkers. In highlighting the contributions of the latter, I aim to demonstrate how the intellectual history of IPE can be told in a less Western-centric manner than is presently the norm in the field. Second, the article also speaks to emerging literature in IPE and other fields that is exploring the new politics of de-globalization in the current era (e.g., Link 2018; van Barneveld et al. 2020). I demonstrate how contemporary advocates of national self-sufficiency are not only invoking similar rationales for this policy as those advanced in the past, but also sometimes citing the historical thinkers analyzed in this article explicitly. I also note, however, how these advocates are forced to recognize the challenges of pursuing ambitious conceptions of autarky in the contemporary global economic context.

## Early European Autarkic Thought

One place to begin an analysis of the history of autarkic thought is with the ideas of Englebert Kaempfer (1651–1716) who developed one of the earliest systematic defenses of autarky in European political economy.^2^ For a theorist of autarky, Kaempfer led a remarkably cosmopolitan life. Born in the Westphalian town of Lemgo, he traveled extensively across Europe and Asia, taking advantage of the growing global economic interconnections of his age. His ideas about autarky emerged from his unusual experience of living in the Dutch settlement at the Japanese port of Nagasaki between 1690 and 1692. Upon his return to Europe in 1692, Kaempfer wrote a book-length manuscript that was published for the first time in full posthumously in an English translation (from the original German) in 1727 under the title *The History of Japan*.^3^ Although unknown to most IPE scholars today, the work became an “immediate bestseller” in Europe at the time and was printed in twelve editions and translations during its first decade alone (Bodart-Bailey 1999, 7). It continued to shape European understandings of Japan until well into the nineteenth century. American admiral Matthew Perry even had a copy with him when he forcibly opened up Japan in 1853–1854 (Mervart 2009).

For those interested in autarkic thought, the importance of Kaempfer's work came in its appendix that had initially been published earlier on its own in Latin in 1712. This portion of his text was devoted to a defense of Japan's decision to become “shut up” without “any Commerce with foreign nations, either at home or abroad” (Kaempfer [1692]1906, 301). Kaempfer exaggerated Japan's economic isolation at the time, but the country's policies had indeed been moving in an autarkic direction since the 1630s when authorities banned Japanese from traveling abroad and restricted all trade with Europe to that involving Dutch merchants in Nagasaki. Kaempfer highlighted three core benefits of Japan's policies, some or all of which he may have picked from Japanese thinkers and officials that he met during his visit.^4^ For our purposes, his arguments were particularly interesting because they anticipated each of the three broad rationales for autarky that would be reiterated by other thinkers throughout the world in subsequent centuries.

First, Kaempfer argued that autarky provided Japanese authorities with autonomy from foreign economic influences in ways that enabled them to pursue whatever domestic policies they wished. As he put it, “they had their hands tied no longer, but were at liberty to do what they thought fit, to attempt things, which it would be impossible to bring about in any open Country, where there is free access and commerce” (Kaempfer [1692]1906, 335). As an advocate for centralized absolutist rule in Europe at the time, Kaempfer placed a high value on this freedom to act for Japanese policymakers (Bodart-Bailey 1995, 4–5). His politics were evident in the kind of policies that he praised the Japanese authorities for pursuing within their autarkic state. These included bringing “towns, burroughs, villages, all colleges and mutual societies ... to the strictest order and regulations imaginable” as well as “appointing multitudes of overseers and rigid censors to have a watchful Eye over the conduct of the people, to keep them within due bounds of submission, to oblige everyone to a strict practice of virtue, and in short, to make the whole Empire, as it were, a school of civility and good manners.” (Bodart-Bailey 1995, 335–36)

Second, Kaempfer highlighted how autarkic policies protected the Japanese from foreign political and cultural influences. He approved of how the country had been “purged of foreigners and foreign customs” because of their disruptive domestic influence (Kaempfer [1692]1906, 330). Indeed, a central rationale for Japan's initial tightening of external controls in the 1630s had been a fear of foreign subversion associated with growing European promotion of Christianity in the country (Laver 2011). Kaempfer argued more generally that all countries that were able to survive without international trade would benefit by keeping their inhabitants away from the “vices” of other countries as well as from their “covetousness, deceits, wars, treachery, and the like” (Kaempfer [1692]1906, 305).

Finally, Kaempfer associated autarky with international peace. He drew this association partly because autarkic policies would insulate countries from foreign influences that encouraged war. He also argued that autarkic countries would have no motivation to go to war. As he put it, countries such as Japan with “all the necessaries of life” internally “should have no reason to entertain any thoughts of invading the rights and properties of others” (Kaempfer [1692]1906, 304). In addition, Kaempfer argued that autarky strengthened Japan's ability to defend itself against external aggression. He praised the “boldness, or Heroism” of the Japanese as well as their “couragious” [*sic*] nature, arguing that these qualities had been encouraged by the kind of society that Japan's self-sufficiency had enabled (Kaempfer [1692]1906, 307, 336; see also Mervart 2009, 327).

In making these arguments, Kaempfer engaged with the free trade views of Dutch natural law philosophers such as Hugo Grotius and Samuel Pufendorf whose ideas he had encountered during his university studies (Bodart-Bailey 1999, 2; Mervart 2009, 2015). Without naming Grotius and Pufendorf directly, he acknowledged that some people would portray the Japanese people as “prisoners” who were “denied all manner of commerce and communication with their neighbours” (Kaempfer [1692]1906, 314). In his view, however, this perspective overlooked the fact that Japan's autarkic policies had left the country “in a happier condition” with inhabitants who were “peaceable,” “virtuous,” and wealthy with “art and industry exceeding all other nations” (Kaempfer [1692]1906, 336). Kaempfer also challenged the Grotian view that international commerce was a force for peace and that supporters of trade restrictions should be accused “of a signal breach of the laws of nature, of an open disregard to the Supreme Will of the All-wise Creator” (Kaempfer [1692]1906, 302). Instead, he argued that it was autarky that was more in keeping with divine will: “Hath not God himself, in that dreadful confusion of tongues at Babel, where men as yet made up one society, given the strongest proofs of his will and intention, that their intimacy and mutual communication should be broke” (Kaempfer [1692]1906, 303).

More generally, Kaempfer acknowledged that foreign commerce enabled a country to obtain the necessities of life as well as useful foreign ideas relating to things such as law, science, religion, mechanical arts, clothing, food, and medicine. But he argued this benefit was not relevant for a country such as Japan which “nature hath proved so very kind to, as to supply it with all these things, necessary for the ease and support of life, and which, through the industry and labour of its inhabitants, hath raised itself to a high pitch of power.” With this argument, Kaempfer highlighted a point that would be raised by many subsequent autarkic thinkers: autarkic policies were more appropriate for some countries than others. As Kaempfer put it, the advantages he outlined were relevant to countries “so long as they can subsist without the produce and manufacturers of foreign Countries” and provided that “such be the state of the Country, as to admitt [*sic*], without any great difficulty, of their being confined within the limits thereof” and that they “have strength and courage enough to defend it [their country], in case of need, against any invasion from abroad” (Kaempfer [1692]1906, 305).

A second important European advocate of autarky was someone familiar with Kaempfer's work: the well-known French thinker Jean-Jacques Rousseau (1712–1778).^5^ Rousseau wrote little about international economic relations in his most famous book *Du Contrat Social* (The Social Contract) published in 1762, but he addressed the subject directly in two later essays he wrote on practical state-building in Corsica (1765) and Poland (1772). In those works, he urged both the Corsican and Polish governments to discourage international trade. Like Kaempfer, he acknowledged the distinctive conditions of each country and tailored his advice accordingly. For Corsica, Rousseau thought almost complete economic self-sufficiency was possible, particularly if the island built a few new factories to produce some necessities that were being imported (Rousseau [1765]2012, 211, 217–19). In Poland, he acknowledged its enduring need for imports of products such as “oil and wine” that could be paid for by limited wheat exports, but urged the following general principle for its economic system: “Think little about abroad, worry little about commerce” (Rousseau [1772]2012, 296, 300).

Like Kaempfer, Rousseau praised how more autarkic policies would provide the Polish and Corsican authorities with autonomy from foreign economic influences. His interest in autonomy, however, reflected quite different domestic preferences from those of Kaempfer. As is well known, Rousseau was critical of the growing commercialization of European societies in his era, a trend that he associated with ills such as inequality, luxury consumption, corruption, immorality, and a loss of individual freedom. In contrast to the French mercantilist policies of Jean-Baptiste Colbert, he favored a decentralized, egalitarian, frugal, agrarian-based economy in which money played a minimal role and in which a democratic republican form of government could flourish. In his view, this kind of domestic order would be undermined by an open trade policy that would force the creation of a more commercially based society. As he told the Corsicans, “If Corsica needed foreigners, she would need money, but since she can be self-sufficient, she does not need any” (Rousseau [1772]2012, 211).

Rousseau also touched on the other two benefits of autarkic policies that Kaempfer had mentioned, although in somewhat different ways. First, Rousseau highlighted how food self-sufficiency would help to insulate countries from foreign political influence: “The only way to maintain a State in independence from others is agriculture. Should you have all the riches of the world, if you do not have the wherewithall to feed yourself you depend on others” (Rousseau [1772]2012, 195). Second, he suggested that autarkic countries would be more peaceful because they were less likely to be attacked than countries that aggressively pursued wealth and power. The latter, he argued, would be a “continual object of greed for the great powers and jealousy for the lesser” (Rousseau [1772]2012, 193). By contrast, his ideal country “will not be held in high esteem abroad, but it will have plenty, peace and liberty at home” (Rousseau [1772]2012, 237). Even if this country did face foreign aggression, Rousseau suggested that its agrarian-focused society would foster strong and patriotic citizens who would be well suited to defend their country (Rousseau [1772]2012, 195, 231, 234, 295).

## Fichte and Other Nineteenth Century European Autarkists

One of Rousseau's intellectual followers, Johann Fichte (1762–1814), then developed the best-known European case for autarky in the nineteenth century in his 1800 book *Der Geschlossene Handelsstaat* (The Closed Commercial State). Although Fichte shared the French thinker's concerns about commercial societies, he rejected Rousseau's preference for agrarian-based economies, arguing that they would only result in “a miserable nation, still half-left-behind in barbarism” (quoted on Nakhimovsky 2011, 112). Instead, he suggested that there was a way to reconcile Rousseau's concerns with a modern economy characterized by an extensive division of labor. His solution was for the state to assume a large role in the domestic economy, regulating employment, wages, and prices in ways that promoted egalitarian goals by guaranteeing citizens the right to work and to live “as agreeably as possible” (quoted in Nakhimovsky 2011, 150). Because rulers might abuse the domestic economic power he wanted them to exercise, he noted that his goals could only be realized in a state committed to republican values.

Fichte argued that autarkic policies were key for his vision because foreign economic influences had to be prevented from disrupting his ambitious conception of government economic activism in the domestic economy. This argument echoed Kaempfer's and Rousseau's, but it put autarky in the service of yet another distinctive domestic goal. Fichte reinforced the case for autarky by arguing that international commerce could be exploitative. In addition to critiquing Europe's “common exploitation of the rest of the world,” Fichte argued more generally that commerce between rich and poor countries was unfavorable to the latter (quoted in Nakhimovsky 2011, 73). Rich countries benefited from positive trade balances, inflows of specie, and higher tax revenue that reinforced their wealth and power. By contrast, poor countries experienced the opposite, resulting in population loss, capital flight, and economies increasingly dependent only on raw material exports. While mercantilist policies might enable a poor country to become more wealthy and powerful, he argued that the success of this strategy could not last if every other state employed it as well. Insulating one's country from international commercial competition altogether was, in Fichte's view, the better solution.

Fichte's conception of autarky went beyond that of Kaempfer and Rousseau in recommending the conversion of all “world” currencies within the country (such as gold and silver) into a new inconvertible state-issued national currency. In addition to strengthening economic control of the border and boosting policy autonomy, this reform would enable the government to manage the domestic money supply to serve national goals. It would also generate funds to invest in initiatives that created domestic substitutes for foreign products. After a transition period, Fichte argued that the only approved trade would consist of bilateral inter-governmental exchanges of surplus goods for imports that compensated for natural differences. Travel abroad would also be controlled, although Fichte encouraged the international movement of scholars and artists whose sharing of ideas and culture would prevent autarkic societies from becoming stagnant (Nakhimovsky 2011). Fichte's commitment to the free flow of ideas and cultural exchange contrasted with Kaempfer's idea that autarky should protect a society from foreign cultural influences.

Like Kaempfer and Rousseau, Fichte also tied autarky to international peace. He lamented how international commerce had become associated with intense European commercial rivalries that led to conflict and colonization. In his view, peace was only possible if these rivalries were eliminated by autarkic policies. Autarky would also mean that countries no longer had any reason to oppress each other since each would have all they needed inside their borders. But Fichte's views on the relationship between autarky and peace also had one important caveat. In the transition period to autarky, Fichte argued that a country such as Prussia might need to annex nearby territories in order to create “natural frontiers” that were more compatible with economic self-sufficiency (quoted in Nakhimovsky 2011, 110). This caveat highlighted the issue Kaempfer had identified: not all countries were perfectly suited for autarky. Fichte saw the redrawing of borders by force as a solution to this problem. He noted that the gold and silver acquired from a country's domestic monetary transformation could be used to hire foreign soldiers for this operation. After this military action, however, Fichte insisted that the “closed commercial state” would renounce any further aggression or acquisition of colonies.

When linking autarky to peace, Fichte also directly challenged the view of some of his liberal contemporaries who believed that international commerce could foster more cosmopolitan identities. Fichte was skeptical of the possibility of the latter: “it seems to me that through our striving to be everything, and to be at home everywhere, we have become nothing, and find ourselves at home nowhere” (quoted on Nakhimovsky 2011, 83). Because commerce encouraged interstate rivalry, he argued that it had instead encouraged unhealthy nationalist sentiments that focused on the link between wealth and national glory. In his view, autarky would foster a healthier national culture: “a higher degree of national honor and a sharply determined national character will develop very quickly. This will be a different, absolutely new nation.” (quoted on Nakhimovsky 2011, 83). He suggested that this new national character, in turn, could lay the groundwork for improved international understanding based on the exchange of ideas and culture rather than commerce.

Three other nineteenth century European thinkers deserve a brief mention because of their distinctive justifications for autarky in this era. One was Adam Müller (1779–1829), a Prussian who worked in Metternich's Austria in the early nineteenth century. Although Müller was initially a fan of Adam Smith, he became increasingly critical of commercial societies from a conservative nationalist standpoint that valued the hierarchical organic unity of feudal agrarian society. To protect the ability of a state to promote this conservative vision, he began to endorse autarkic policies. He also emphasized how these policies would reinforce peoples’ tie to their nation, particularly if they included an inconvertible national currency which could serve as an expression of the nation's “inner spiritual unity” (quoted in Pribam 1983, 212; see also Harada 2001). While participation in the international economy undermined a national identity, autarky would reinforce it.

One of the leading French protectionists in the 1840s, the French industrialist Auguste Mimerel (1786–1871), developed a quite different case for autarky. He worried about the impact of international competitive pressures on the livelihood of French industrial workers. In his view, the wages of French industrial workers were being driven down by competition from Britain where he argued workers were subject to “debasement” and exploitation by British elites. For Mimerel, autarky was a means of protecting France's more egalitarian “social organization and customs” from these foreign economic pressures (quotes in Todd 2015, 170, 169)

Yet another case for autarkic policies was developed by the conservative German economist Karl Oldenberg (1864–1936) near the end of the nineteenth century. Like Mimerel, Oldenberg worried that the wages of domestic workers were being forced down by international competitive pressures (including from low wage countries such as Japan and China). But as a critic of Germany's increasingly industrial society, he was also concerned that profitable export industries were drawing Germans out of agriculture in ways that undermined the country's rural culture and left it dependent on food imports and uncertain export markets. As he put it, “we can live without industry, but not without food” (quoted on Lebovics 1967, 44). Oldenberg also noted Germany's growing economic vulnerability in a world where major powers such as the United States, Russia, and perhaps even Britain were moving in a protectionist direction. If Germany lost its export markets, he argued that rising unemployment would reinforce domestic class conflicts that industrialization had already generated. Imported food might also become scarcer, particularly as many other countries industrialized (Barkin 1970, 150–74).

For all these reasons, Oldenberg recommended that Germany pursue “self-sufficiency” as a long-term goal in order “that we remain masters in our own house” (quoted in Lebovics 1967, 45). He acknowledged that an abrupt turn to national autarky was unrealistic for a country such as Germany that was deeply integrated in the increasingly globalized economy of the late nineteenth century. However, he hoped that Germany could gradually cultivate greater self-sufficiency—particularly “*Nahrungsautarkie*” (food autarky)—via trade protectionism and domestic policies that encouraged Germans to rebuild their country's traditional rural, agrarian economy (quoted in Lebovics 1967, 51). Oldenberg also differed from Kaempfer, Rousseau, and (more ambiguously) Fichte in downplaying the link between autarky and peace. Referring to Germany's policy at the time of pursuing global power and imperial expansion, he argued that his autarkic vision would not involve a “repudiation of *Weltpolitik*, of a strong fleet, or of colonies” (quoted in Lebovics 1967, 45).

## Northeast Asian Autarkists

Although the history of political economy is usually told in a Western-centric manner, this approach has been attracting growing criticism. Its limitations are particularly evident in the case of the history of autarkic thought where many important contributions were made by thinkers outside the West. Take, for example, the case of Japan. We have already seen how Kaempfer's important views may have been influenced by his encounters with Japanese thinkers in the 1690s who defended their country's policies at the time. When Japanese authorities embraced even more autarkic policies in the late eighteenth and early nineteenth centuries, Japanese scholars justified this policy in some interesting ways.

Particularly important at the turn of the nineteenth century were the ideas of the scholar Shizuki Tadao (1760–1806) who coined the phrase *sakoku* (national isolation) in 1801 to describe the country's policies (Mervart 2009, 2015). At the time, Japanese officials and thinkers were increasingly concerned about the growing presence of British and Russian merchants in their region. Some Japanese urged the adoption of new outward-oriented mercantilist policies to deal with the growing foreign threat, but Shizuki praised *sakoku*’s role in defending the country's cultural and economic autonomy, arguing that it protected Japan from “having our customs disturbed and our fortunes plundered by foreigners” (quoted in Hiroshi 2006, 20). The reference to foreign plundering evoked earlier Japanese concerns that foreigners were robbing the country of its wealth by selling them useless goods in exchange for gold and silver from Japanese mines that could never be replenished.^6^

Interestingly, Shizuki's defense also included a translation of the appendix of Kaempfer's history of Japan that had earlier praised Japan's autarkic polices. Indeed, the phrase *sakoku* came from the title Shizuki chose for this translation: *Sakoku-ron* (Theory of National Isolation). Shizuki told his readers that he hoped his translation would help to stimulate a sense of national pride and identification—what he called “national gratitude”—arising from living in a country with all of the virtues outlined by the foreign author (quoted in Hiroshi 2006, 20). To the best of my knowledge, Kaempfer's work was the first European work addressing political economy to be translated into Japanese (and over eight decades before translations of *The Wealth of Nations* or List's best known work *The National System of Political Economy*). To the extent that Kaempfer's arguments drew on earlier Japanese ideas, Shizuki's translation also represented a remarkable example of how autarkic ideas flowed from East to West and back again at this early historical moment.

One of the themes in Shizuki's work was developed in more detail in 1825 by the prominent Japanese thinker Aizawa Seishisai (1782–1863). Shizuki had noted that the growing foreign threat could play a useful role of “stiffening the most urgent resolve for defense against external threats and harmony at home” (quoted in Hiroshi 2006, 20). This idea was at the center of Aizawa's work *Shinron* (New Theses) that defended a government order in 1825 calling for the use of force to repel foreign ships attempting to land in Japan. Aizawa praised how the government's new policy might provoke a conflict with foreigners that would “unify the will of the people” through a reassertion of traditional Confucian values in ways that could enable Japan fend off the foreigners (quoted in Hiroshi 2006, 23). Instead of seeing autarky as a force for peace, Aizawa hoped it might provoke war.

Aizawa advanced three other arguments in support of autarky. First, he repeated past Japanese concerns about foreign political influence, noting that international trade provided foreigners with opportunities to promote Christianity in ways that undermined the loyalty of the Japanese to their country and might serve as a prelude to annexation (Wakabayashi 1986, 86–90, 109, 112). He also echoed longstanding Japanese criticism of the exploitative nature of international commerce, arguing that “[f]oreign trade is largely a frittering away of our precious metals for useless commodities” (Aizawa [1825]1986, 239). Finally, he saw autarky as a way to challenge the growing commercialization and decadence of Japanese society and to restore Japan's traditional agrarian-focused economy. For Aizawa, imports were largely “luxury items” while exports encouraged cash crop agriculture that undermined traditional Japanese farming and domestic food needs (Aizawa [1825]1986, 239). Aizawa's desire to protect Japanese society from foreign commercial pressures bore some similarities to Rousseau's ideas (which he did not know), but quite different was his goal of building a society around what Bob Wakabayashi (1986, 6) calls a kind of “muscular Confucianism.”

The works of Shizuki and Aizawa initially circulated only privately in intellectual circles, but both were finally published in the 1850s in the context of the heated debates about the country's economic opening at the time.^7^ Shizuki's phrase *sakoku* quickly became widely used to describe Japan's past foreign economic policy, while Aizawa's text became “a virtual bible” to activists in the 1860s who were committed to expelling foreigners from Japan after Perry's arrival (Wakabayashi 1986, ix). Aizawa himself, however, had decided by this time that Japan needed to accept economic opening via treaties with the foreign powers in order to avoid invasion and colonization (Wakabayashi 1986, 24, 135–37). After the 1868 Meiji Restoration, most Japanese thinkers also began to accept the country's integration into world markets, with many urging the adoption of activist, neomercantilist policies to boost Japan's wealth and power in the increasingly integrated global economy of their era.

But a few prominent supporters of autarky remained. The best known in the 1870s and early 1880s was the Buddhist public intellectual Sada Kaiseki (1818–1882) who led boycotts across the country of foreign products, such as Western kerosene oil lamps, umbrellas, hats, railways, steamboats, soap, and wine (Rambelli 2011, 2017). Like other autarkists, Kaiseki argued that his country needed to be protected from foreign influences, but his specific aspirations for his country were quite innovative. Criticizing the Western model of industrialization being embraced by the country's leaders at the time, Kaiseki suggested that Japan needed economic policies that were better suited to Japan's distinctive culture and economic needs. Instead of generating material progress by Western-style, machine-based mass production, he urged a strategy based on agriculture and Japanese artisan methods of production, which were more human scale and in tune with nature. His ecological sentiments were also evident in his opposition to foreign products that relied on fossil fuels whose supply was finite. In his words, carbon “is not recreated” once it is used (quoted on Rambelli 2011, 122).

Kaiseki argued that autarky was needed for his preferred model of economic development to succeed. Cheap and attractive foreign imports risked undermining the local artisan producers that were central to his economic strategy. They also introduced foreign cultural influences that shifted local tastes away from locally produced goods. In addition, he was concerned that new export-oriented monocrop production was causing soil exhaustion and deforestation that threatened to undermine the country's long-term prosperity. Instead of relying on foreign demand and the promotion of Western-style industry, he argued that public authorities should promote consumption of locally made agricultural and artisan products, even if this resulted in some economic inefficiencies. As he put it, “inconvenience must be esteemed” (quoted in Sugiyama 1994, 1).

Kaiseki's boycott campaign had limited results in Japan, but support for autarky was stronger at this time in neighboring Korea which refused economic opening until the mid-1870s. Dating back to the seventeenth century, Korea had maintained a policy of seclusion vis-à-vis the West that was often stricter than that of Japan. As foreign pressure for economic opening grew after the mid-nineteenth century, however, some Koreans began to urge the embrace of a more outward-oriented, neomercantilist economic strategy to address the new external challenge (Helleiner forthcoming, Chapter 9). In this context, supporters of the traditional autarkic policy were increasingly forced to justify it more explicitly.

They were initially led by the prominent scholar Lee Hang-ro (1792–1868) who had been specially recruited by the government to devise policies for dealing with Western powers (Chung 1995). Lee was most worried about how economic opening would leave the country vulnerable to foreign cultural influences that undermined its neo-Confucian society. He argued that international trade would encourage profit-seeking behavior and an inflow of Western materialist ideas and consumer goods that threatened the country's neo-Confucian values such as frugality and modesty. In his view, economic openness would contaminate the minds of the Korean people by unleashing a “flood” of unhealthy “human desires” that reduced them to “barbarians and beasts” (quoted in Chung 1995, 126–27). Lee's concern was summed up in his warning phrase “exchange of commodities, exchange of immorality” (*tonghwa tongsaek*) that became a dominant theme of opponents of economic opening in the late nineteenth century (quoted in McNamara 1996, 62).

Lee also shared some of the economic and political concerns of other autarkists. In the economic realm, Lee argued that the country's opening would undermine traditional Korean producers who were exposed to new foreign competition. He also suggested that international commerce would exploit Korea economically because it would be exporting resources whose supply was limited in exchange for foreign manufacturers that could be produced “without restriction” (quoted in McNamara 1996, 208). Politically, Lee predicted that economic opening would expose Korea to dangerous foreign political influence within the country. By contrast, he suggested—like Kaempfer and Rousseau—that autarky would strengthen Korea's ability to defend itself against foreign aggression. In Chai-sik Chung's (1995, 209) words, this argument stemmed from Lee's belief that “[e]xternal defence of the state and society was contingent upon internal reform—the internal cultivation of the self.”

## Other Autarkists beyond the West

Prominent advocates of autarky existed in other non-Western regions of the world as well. The best known example in the nineteenth-century Latin America was José Gaspar Rodríguez de Francia (1766–1840), the ruler of Paraguay from 1814 until his death in 1840. After organizing and being elected by what Richard White (1978, 56) calls “Latin America's first popular congress,” Francia set out to create what he termed a “self-sustaining” economy without much dependence on the outside world (quoted in White (1978, 125). To this end, he fostered some manufacturing to reduce import needs and encouraged the country's export-oriented agricultural sector to focus on the domestic market. All external trade was channeled through two locations, where some of the country's surplus commodities were traded by barter for goods Paraguay could not produce (Whigham 1991; White 1978; Williams 1979, Chapter 4). Francia's efforts to create what he called “a system of non-intercourse” with the outside world were not entirely successful, but Paraguay did become the only country in South America to produce almost all of own clothing at a time when others were experiencing growing imports of cheap industrially produced British products (quoted in Robertson [1939]1970a, 279; see also Williams 1979, 92; Batou 1990, 243)^8^

Francia's tight controls on external trade earned Paraguay a reputation as “inland Japan” under his leadership (Whigham 1988, 279). However, Francia's intellectual influences did not come from Japan. Instead, he had what one scholar calls a “Rousseauan view of life” that he picked up during his university education that culminated in a PhD in theology from Argentina's University of Córdoba in 1785 (Williams 1979, 20). In addition to emphasizing the need for an agrarian-based economy, Francia promoted Rousseauan goals such as frugality, egalitarianism, patriotism, and especially the protection of his country's sovereignty from foreign political influence and “dependency” (quoted in White 1978, 138; see also Robertson 1939]1970a, 279–80; Williams 1979, 78).

In the Caribbean a few decades later, another supporter of autarky was Edmund Paul (1837–1893) who emerged as a prominent politician in the 1870s and “was one of the most significant political thinkers in nineteenth-century Haiti” (Nicholls 1996, 102). In the early 1860s, Paul published an analysis suggesting that his country work toward Fichte's closed commercial state as a long-term goal. While many in Haiti were content with the country's role as an agricultural exporter, Paul wanted Haiti to move in a self-sufficient direction by promoting local industry through tariffs and other forms of government support. In his view, Haiti's political independence required economic independence that could only be cultivated through this economic transformation (Nicholls 1996). To avoid foreign influence, Paul also argued that Haiti's economic development should be financed as much as possible by local capital and he criticized the lack of patriotism of Haitians who exported their money to Europe. In addition, he endorsed an existing ban on foreign ownership of land, arguing that it protected the small local landholders against an influx of foreign investors. The latter, he feared, would create a kind of economic slavery similar to the legal slavery against which the Haitian revolution had triumphed. As he put it, “To accord the right of property to whites while color prejudice is still prevalent, would be to renounce the end to which the nation pursues.” He warned that throughout the world “up to this day, the prosperity of whites is founded on the degradation of the blacks” (quotes in Nicholls 1996, 103).

Turning to Africa, a particularly interesting advocate of autarky was Kobina Sekyi (1892–1956) who has been described as “one of the most outstanding intellectuals in West Africa, and indeed in colonial Africa in general” (Langley 1979, 44). Born in the British colony of the Gold Coast, Seyki studied philosophy at the University of London after which he became a prominent conservative anti-colonial nationalist. Sekyi ([1917]1979, 244) was particularly critical of European imperialism for “denationalizing” the peoples it conquered and he urged Africans to insulate their cultures and nationalities from European civilization. The latter was, in his view, “based on commerce or trade” in ways that undermined natural social ties, generated inequality and greed, and resulted in “over-luxurious,” “diseased” societies (Sekyi ([1917]1979, 248, 243).

In Sekyi's view, Africans needed to pursue development strategies that reflected their own culture: “[l]et each social group develop along the lines marked out for them by their unwesternized and therefore undemoralised ancestors, accepting from the West only such institutions as can be adapted to, and not such as cannot but alter, their national life” (Sekyi ([1917]1979, 250). To meet those goals, Seyki argued that future independent African countries needed to embrace economic autarky, engaging in international trade only by barter when necessary. The pursuit of this autarkic strategy would, he argued, also contribute to world peace. His reasoning was that the alternative strategy of following Europe's model would lead Africa to “ever creating new wants to supply an insatiable desire for conquest, ever oppressing others to further this conquest, and bound to end by consuming all that has been acquired by such conquest in universal holocaust kindled by the demon of Greed” (Sekyi ([1917]1979, 244–45).

In South Asia, the most famous advocate of autarky was Mohandas Karamchand Gandhi (1869–1948). Although Gandhi is best known for his lead role in the Indian independence struggle, he combined his anti-colonial politics with a compelling economic vision of *swadeshi*—or economic self-sufficiency—for India. He wanted to see his country build upon its ancient tradition of self-governing villages whose economies were focused on localized artisan and agrarian activities, and where people were not caught up in “the modern rush, and the multiplication of wants and machinery to supply them” (quoted on Ganguli 1977, 251). Gandhi's vision contrasted with the economic views of other Indian nationalists such as Jawaharlal Nehru who favored a neomercantilist, state-led industrialization strategy to catch up to the wealth and power of Western industrial countries. In Gandhi's view, that strategy overlooked the drawbacks of modern industrial Western civilization with its extreme inequalities, exploitation, and violence that were incompatible with individual freedom and democratic life (Trivedi 2007).

For his economic vision to be realized, Gandhi recognized the need to insulate India from foreign economic influences through autarkic policies. Gandhi highlighted how imports from imperial Britain had undermined traditional Indian producers and drained wealth from India (Gandhi 1997, 168; 1971, 158, 239). Drawing on this experience of colonial exploitation, he argued: “It is certainty our right and duty to discard everything foreign that is superfluous and even everything foreign that is *necessary* if we can produce or manufacture it in our country” (Gandhi 1971, 262). Gandhi also suggested that imports would not be needed because “every village of India will almost be a self-supporting and self-contained unit exchanging only such necessary commodities with other villages where they are not locally producible” (quoted on Gupta 1968, 143). In addition, Gandhi argued that Indian producers would not feel any need to export because local consumers would provide a stable market for their goods (Ganguli 1977, 247).

In outlining this vision, Gandhi sometimes countered liberal arguments for free trade directly. For example, when critics noted that homespun goods would be more expensive, Gandhi replied that economics was not just about economic efficiency and material progress but also about ethics and spirituality (Gerth 2003, 18). He insisted that the kind of material economic progress discussed by Western economic thinkers and valued by Western civilization was “antagonistic to real progress” of a moral kind. He called on India to resist “the monster-god of materialism” that had “stunted” the West's “moral growth” and instead to become a “truly spiritual nation” by building on ancient ideals (Gandhi 1997, 160, 162). In this sense, his case for autarky echoed that of some other non-Western thinkers who were concerned not just about foreign influences of an economic kind but also cultural ones.

Gandhi also challenged the equation drawn by many liberals between free trade and peace. In his view, “[m]ost of the wars of our times spring from greed for money” (Gandhi 1971, 371). He argued that Western industrial civilization generated excess production that encouraged the pursuit of export markets and free trade, including through violence, exploitation, and war. If his country emulated Western civilization, Gandhi—like Sekyi—argued that it would become a threat to world peace: “England with her large-scale production has to look for a market elsewhere. We call it exploitation. And an exploiting England is a danger to the world, but if that is so, how much more so would be an exploiting India, if she took to machinery and produced cloth many times in excess of requirements” (Gandhi 1971, 47).

If *swadeshi* economics would prevent India from becoming aggressive toward other countries, Gandhi acknowledged that some people might question whether it would be able to defend itself sufficiently “in the face of a world armed to the teeth” (quoted on Ganguli 1977, 253). Like Lee Hang-ro in Korea, however, he suggested that people living in his ideal state would be well equipped to defend themselves because of their moral strength: “If we will but clean our houses, our palaces and temples of the attributes of wealth and show in them the attributes of morality, we can offer battle to any combinations of hostile forces without having to carry the burden of a heavy militia” (Gandhi 1997, 162). He also argued that a “[r]urally organised India will run less risk of foreign invasion than urbanised India well-equipped with Military, Naval and Air Forces.” His reasoning echoed that of Rousseau: “Simple homes from which there is nothing to take away require no policing” (quoted in Agarwal 1944, 21).

## The Popularity of National Self-Sufficiency in the 1930s

It was in the 1930s that autarkic thought achieved its greatest political influence across the globe. The trigger was the Great Depression of 1929–1933 and the associated collapse of cross-border trade, foreign lending, and the international gold standard. As international economic relationships unraveled, many countries—especially debtors—reinforced the de-globalization trend by imposing tight controls on cross-border economic activity as a way of coping with the economic shock. This turn to more autarkic policies was supported and reinforced by a number of thinkers.

The most famous of these was British economist John Maynard Keynes (1883–1946) in his 1933 essay “National Self-Sufficiency.” Because Keynes had already become an international celebrity, his essay attracted enormous attention and was published in outlets in Britain, Ireland, the United States, and Germany in that year alone.^9^ It remains the best-known autarkic tract of the twentieth century. Keynes had endorsed free trade in his youth, but he turned his back completely on these earlier views in this essay, insisting that it was necessary now “to shuffle out of the mental habits of the prewar nineteenth-century world” (Keynes 1933, 755). At the same time, his vision of national self-sufficiency was not absolute. It focused on reducing the flow of goods and especially finance, while still allowing for some cross-border flows:

I sympathize…with those who would minimize, rather than with those who maximize, economic entanglement among nations. Ideas, knowledge, science, hospitality, travel - these are the things which should of their nature be international. But let goods be homespun whenever it is reasonably and conveniently possible, and above all, let finance be primarily national. (Keynes 1933, 758)

Keynes’ emphasis on financial autarky and his insistence on the enduring flow of ideas were reminiscent of Fichte. His core case for autarky also bore some similarities to the earlier German thinker's emphasis on the need for policy autonomy to pursue domestic economic activism. In Keynes’ view, governments needed to be freed from external constraints in order to try new kinds of economic policies to address the economic crisis: “we all need to be as free as possible of interference from economic changes elsewhere, in order to make our own favorite experiments towards the ideal social republic of the future.” He continued: “a deliberate movement towards greater national self-sufficiency and economic isolation will make our task easier, in so far as it can be accomplished without excessive economic cost” (Keynes 1933, 763). Keynes himself favored activist macroeconomic policies aimed at promoting full employment. For these policies to be effective, he noted that greater national self-sufficiency—particularly controls on financial movements—would allow Britain to lower its interest rate in ways that generated higher “material prosperity” for the country (Keynes 1933, 763).

Like some earlier autarkists, Keynes also highlighted his broader distaste for overly commercially oriented societies. He criticized the kind of “decadent international but individualistic capitalism” [760–61] in place during the 1920s, arguing that “[i]t is not intelligent, it is not beautiful, it is not just, it is not virtuous—and it doesn't deliver the goods” (Keynes 1933, 761). He also attacked the nineteenth century liberal focus on “financial results” at the expense of other values, including equity and environmental goals:

The whole conduct of life was made into a sort of parody of an accountant's nightmare. Instead of using their vastly increased material and technical resources to build a wonder city, the men of the nineteenth century built slums; and they thought it right and advisable to build slums because slums, on the test of private enterprise, “paid” … We destroy the beauty of the countryside because the unappropriated splendours of nature have no economic value. We are capable of shutting off the sun and the stars because they do not pay a dividend. (Keynes 1933, 763–64)

While endorsing the need for domestic political-economic “experiments,” Keynes also made clear that he had no sympathy for those being pursued in places such as Hitler's Germany or Stalin's Russia. Writing soon after Hitler came to power, he described Germany as “at the mercy of unchained irresponsibles” (Keynes 1933, 766).^10^ He also attacked the Russian government for its “sacrifice of almost everything that makes life worth living to wooden heads,” noting that “Stalin has eliminated every independent, critical mind, even those sympathetic in general outlook” (Keynes 1933, 766, 769). In keeping with his liberal domestic values, Keynes insisted that autarky should never coexist with “Intolerance and the stifling of instructed criticism” and highlighted his own preference for a domestic economic order that retained “as much private judgment and initiative and enterprise as possible” (Keynes 1933, 762, 768).

Keynes acknowledged a policy of national self-sufficiency might be too costly for small countries such as Ireland that would lose the economic benefits of international trade.^11^ For larger countries, however, Keynes thought those benefits were overstated because “most modern processes of mass production can be performed in most countries and climates with almost equal efficiency” (Keynes 1933, 760). In high-income countries, he also noted tradeable “primary and manufactured products play a smaller relative part in the national economy compared with houses, personal services, and local amenities, which are not equally available for international exchange” (Keynes 1933, 760). More generally, he argued that the costs of abandoning free trade would be more than offset by the economic gains arising from domestic economic activism made possible by “gradually bringing the product and the consumer within the ambit of the same national, economic, and financial organization” (Keynes 1933, 760).

Like some past autarkic thinkers, Keynes also suggested that this policy might be a force for peace. Challenging the liberal equation between free trade and peace, he suggested that economic interdependence might generate more, rather than less, reasons to go to war: “The protection of a country's existing foreign interests, the capture of new markets, the progress of economic imperialism—these are a scarcely avoidable part of a scheme of things which aims at the maximum of international specialization and at the maximum geographical diffusion of capital wherever its seat of ownership” (Keynes 1933, 757). He also noted that the historical record called into question the liberal case: “the age of economic internationalism was not particularly successful in avoiding war” (Keynes 1933, 758).

Although Keynes’ 1933 article was the most high-profile intellectual defense of autarky during the early 1930s, it was not the only one. In 1931 and 1932, a German conservative writer and Nazi supporter, Friedrich Zimmermann (aka Ferdinand Fried) (1898–1967), published two books titled *Das Ende des Kapitalismus* (The End of Capitalism) and *Autarkie* (Autarky) that advocated “giving up the idea of the world economy” in favor of “the isolation of individual national economic spheres” (quoted in Szejnmann 2013, 363). Described by Stefan Link (2018, 359) as “the foremost proponent of autarky in Depression Germany,” Zimmermann argued that this policy would protect the German state from foreign political influence and enable the introduction of a planned economy involving the “total renunciation of capitalism and liberalism” (quoted in Szejnmann 2013, 363). It could also allow Germany to throw off the exploitative burden of the country's crushing external debt burdens. As Zimmermann put it, “[t]he entire compulsion of international debt and interest payments holds sway only as long as individual countries are compelled to be members of the global economy” (quoted in Link 2018, 359). In addition, Zimmermann echoed Müller in arguing that autarky would become a “conscious expression” of Germany's national will (quoted in Braatz 1971, 579).^12^

When advocating autarky, German fascists envisioned an “extended economic space” that was inclusive of Central and Southeastern Europe, a region that they hoped would provide Germany with the resources and agricultural produce it needed (quoted in Szejnmann 2013, 361). Before invading the region in the later 1930s, the Nazi government manipulated bilateral economic relationships with Central and Southeastern European countries through neomercantilist policies that fostered their economic dependence on Germany (Hirschmann 1945). In some ways, the subsequent German conquest of the region was consistent with the ideas of Fichte who had endorsed the annexation of neighboring countries to create a territory in which economic self-sufficiency was possible. However, Fichte's vision that the resulting “closed commercial state” would be peaceful was not echoed in German fascist thought. The Nazis saw their country engaged in an endless social Darwinian struggle for survival in which further conquest and domination was needed (Szejnmann 2013, 246).

The same was true of fascists elsewhere, including those in Italy whose military expansion into north Africa in the mid-1930s was driven by similar ambitions. Even before the onset of the Great Depression, Mussolini's government had become interested in autarkic policies in the late 1920s as a way to strengthen Italy's political sovereignty and gain economic autonomy to pursue increasingly ambitious forms of domestic planning (e.g., Gregor 2005, Chapter 6; Woolf 1968). Japanese fascists in the 1930s also combined their embrace of autarkic ideas with support for military expansion to create a larger closed economic space that became called in 1940 the “Greater East Asia Co-Prosperity Sphere.” As Stuart Woolf (1968, 140) puts it, “[t]he creation of the empire was the logical development of the conviction that the country needed to isolate itself from the world .... the empire formed an essential part in the plan to achieve independence within a closed economy.”.

The autarkic policies of these leading fascist powers also inspired thinkers elsewhere in the 1930s. For example, in the early 1930s, prominent figures in the Nationalist government in China, such as Wang Jingwei (1883–1944) and Chen Gongbo (1892–1946), were attracted to the economic policies of Italian fascism, including its embrace of autarky which they saw as a policy of “nation-building” (quoted in Zanasi 2006, 47). Internally, Wang and Chen argued that autarky would boost the nation's political and economic cohesion by forcing the industrializing coastal regions to reorient inwardly to sell to the rural economy and draw resources from it. In their view, autarky would also insulate China from external political interference and strengthen its ability to resist Japanese imperialism. The anti-imperialist orientation of this autarkic vision contrasted with that of many European and Japanese fascists (Zanasi 2006, 14).

The same anti-imperialist orientation characterized a group of left-wing Turkish thinkers led by Şevket Süreyya Aydemir (1897–1976) who advocated for more autarkic policies in the early 1930s. In the context of a collapsing liberal international economic order, they argued that Turkey should embrace a high degree of national self-sufficiency as a way of ending its position as one of the exploited, agricultural-producing “semi-colonies” in the world economy (quoted in Barlas 1998, 49). For them, Turkey's political independence in the 1920s had to be matched in the 1930s by the creation of real economic independence of this kind. While allowing for some international trade in specialized goods, they urged a reorientation of production toward internal markets as well as state economic planning to promote ambitious industrialization goals. Although attracted to authoritarian politics, they were critical of fascist goals, including the endorsement of imperialism and the exploitation of colonial peoples by European fascist powers (Barlas 1998, 47–50; Hanioğlu 2011, 188–91; Türkeş 1998).

## Autarky in the Post-1945 World: From Rejection to Revival?

While the popularity of autarkic thought reached a highpoint during the 1930s, it subsequently dropped dramatically. The architects of the post-1945 international economic order explicitly rejected this ideology by committing to economic multilateralism and openness at the 1944 Bretton Woods conference. Among them was Keynes who turned his back on his 1933 views. By the early 1940s, he became committed to building a new kind of multilateral economic order that enabled the kinds of activist economic policies he favored. His “embedded liberal” philosophy was shared by many other participants in the Bretton Woods negotiations, including key American officials (Ruggie 1982).

One reason why the Bretton Woods delegates rejected autarkic policies was their belief that the goal of national self-sufficiency had been discredited by the experience of the 1930s. The turn to autarky was blamed economically for exacerbating the Great Depression. It was also tarnished by its association with fascism and the decline of international cooperation that resulted in war. As the leader of the French delegation Pierre Mendès-France put it at the end of the 1944 conference, his government was “[o]pposed to autarchy” and “to all techniques consistent with the preparation, the continuation or the liquidation of a war, but inconceivable in a world guided by fraternal cooperation of all people of good will” (US Department of State 1948, 1115). The association of autarky with conflict and war overlooked the fact that many autarkists in the past—including Keynes—had seen the policy as a force for peace, but it made sense as a reaction against the specific fascist strand of autarkic thought of the 1930s.

Autarkic ideals remained unpopular in most parts of the world during the first few decades of the postwar period. In Western countries, support for international economic openness remained high in the context of the influential normative framework of embedded liberalism. When the Soviet Union and its allies rejected this order with the onset of the Cold War in the late 1940s, they too created an economically interdependent bloc, but one run on socialist principles.^13^ When decolonization accelerated, policymakers in most newly independent countries were more attracted to neomercantilist ideas and policies than autarkic ones. To be sure, some declared their interest in greater national “self-reliance” during the 1960s and 1970s, but this idea usually referred only to a partial scaling back of economic relations with the West, while expanding economic ties with other lower-income countries and the socialist bloc (e.g., Biersteker 1980). Even Marxist dependency theorists who urged countries to “de-link” from global capitalism insisted that they were not advocating autarky (e.g., Amin 1985, 11, 18, 62).

Of course, there were some exceptions where policymakers embraced autarkic ideology. One was North Korea whose authoritarian leader, Kim Il-sung (1912–1994) declared his country's official state ideology to be *juche* in 1972. This ideology emphasized the interconnections between economic autarky, political and ideological independence, and military self-defense (Lee 2003). Some scholars suggest parallels between *juche* and Korea's pre-1870s isolationism as well as its country's neo-Confucian ideas of that earlier time (David-West 2011; Kang 2011; e.g., Armstrong 2013, 82). One key difference, however, was that Kim was committed to ambitious goals of socialist industrial developmentalism rather than the conservative feudal agrarianism of Lee Hang-ro.

A more systemically significant example came from China after Mao Zedong's (1893–1976) break with the Soviet Union in the late 1950s. In the context of this political rupture and the ongoing Western economic embargo of China, Mao extolled the benefits of “self-reliance”—or *zili gengsheng—*as a means of minimizing China's economic and political dependence on foreigners and avoiding exploitation by both the Soviet Union and capitalist powers (Wu 1981; Kerr 2007). This Chinese phrase had a literal meaning of “regeneration by one's own efforts” and Mao had used it in a broader way as far back as the late 1930s. As David Kerr (2007, 81) puts it, it could imply simply that “the responsibility for China's development, both in theory and practice, should lie with the Chinese themselves.” In this period, however, the concept took on the meaning of fostering national economic self-sufficiency.

If support for autarky was limited in the early postwar decades, it became even more so after 1980 in the context of intensifying global economic integration, the growing popularity of neoliberal ideas, and China's dramatic economic opening after Mao's death. Beginning in the mid-1990s, however, ideas of national self-sufficiency began to attract new supporters, some of whom even invoked earlier autarkists explicitly. In the anti-globalization movements that became prominent in the late 1990s, some thinkers cited Keynes’ 1933 article and Gandhi's vision of *swadeshi* as sources of inspiration (e.g., Kumar 1996; Daly and Cobb 1994, Chapter 11; Lang and Hines 1993, 28). The latter was particularly popular among supporters of increasingly influential “green” ideology that criticized the social and ecological costs of large-scale industrial economies. In a similar way as Gandhi, green theorists and activists called for the restoration of vibrant local self-reliant communities that carved out some autonomy from pressures of global cultural homogenization as well as from foreign economic exploitation and competition (e.g., Mander and Goldsmith 1996).

Gandhi's thought was also invoked by a new “food sovereignty” movement that emerged in this same period in many countries calling for national agricultural policies to “prioritize production for domestic consumption and food self-sufficiency” (La Via Campensina [1996]2010, 198; see for the use of Gandhi, see Glaab and Partzch 2018). This movement has also emphasized the importance of insulating local communities against foreign economic and cultural influences. Interest in ideas of food self-sufficiency then widened in the wake of the 2008 global financial crisis. The sudden volatility of world food prices at the time, combined with unilateral bans on food exports, left many policymakers concerned about their countries’ dependence on imported food and their vulnerability to international market trends and foreign political decisions. In this context, many governments began to give new priority to the goal of increasing food self-sufficiency and some even formally endorsed the idea of “food sovereignty” in official documents, such as those in Bolivia, Ecuador, Mali, Nepal, Nicaragua, Senegal, and Venezuela (Clapp 2017, 92; Hamilton-Hart 2019).

The election of Donald Trump as American president in 2016 encouraged further interest in ideas of national self-sufficiency. After coming to power, the Trump administration took initiatives to reduce America's dependence on global supply chains, partly to insulate American workers from foreign competition and partly for the national security reason of reducing the “level of foreign dependence on competitor nations” (U.S. Department of Defense 2018, 3). These goals built upon anti-globalization sentiments within American society that had been growing since the 1990s and especially since the 2008 financial crisis. Trump's worldview was much closer to a neomercantilist one than an autarkist one, but some of his supporters on the far right are more clearly in the latter camp.^14^ For example, in a 2020 publication from the Claremont Institute, Curtis Yarvin called for the promotion of an “isolationist” policy of “neo-Sakoku”. Like some other past autarkists, he argued that a world of autarkic states would be more peaceful because the reasons for conflict would diminish (Yarvin 2020).

The Trump administration also indirectly encouraged new interest in greater national self-sufficiency in other countries because of its protectionism and its broader “weaponization” of America's international economic relations (Farrell and Newman 2019). Both its economic policies and the rising geopolitical tensions accompanying them prompted foreigners to recognize their vulnerability to foreign political influence arising from economic interdependence. This recognition has been particularly evident in China, the country that has been a central target of the Trump administration's policies. In the fall of 2018, President Xi Jinping even began to resurrect Mao's idea of *zili gengsheng*, arguing that it needed once again to become the foundation of Chinese policymaking because of international developments. As he put it, “Unilateralism and trade protectionism have risen, forcing us to travel the road of self-reliance (quoted in Wildau 2018).

If interest in greater national self-sufficiency was rising before 2020, the COVID-19 crisis gave the idea much greater political salience. The crisis generated a rapid decline in cross-border economic flows similar to the de-globalization trend of the early 1930s. The sudden closure of borders also forced businesses, governments, and citizens to recognize in a much more serious way how economic interdependence left them vulnerable to international markets and the actions of foreign governments. Even as they focused on short-term crisis management, policymakers began to emphasize the need to boost national self-sufficiency over the medium term in order to strengthen their jurisdictions’ economic resilience to future shocks.

Take, for example, India's Prime Minister Narendra Modi who committed to create a “self-sufficient India” as a new national goal in a major speech in May 2020. Previously a champion of globalization, Modi now argued that “[t]he state of the world today teaches us that a “Self-reliant India” is the only path.” After describing India's successful development of new manufacturing capacity for N-95 masks and personal protective equipment during the crisis, he noted the following: “the Corona crisis has ... explained to us the importance of Local manufacturing, Local market and Local supply chain. In times of crisis, this Local has fulfilled our demand, this Local has saved us. Local is not just the need, it is our responsibility also. Time has taught us that we must make the Local as a mantra of our life ... from today every Indian has to become vocal for their local, not only to buy local products, but also to promote them proudly.” Modi also followed some past autarkic thinkers in tying his goals to the cause of world peace: “India's self-reliance is ingrained in the happiness, cooperation and peace of the world” (Modi 2020).

Other policymakers have also expressed their desire for greater self-sufficiency since the start of the pandemic. For example, in the same month as Modi's speech, the European Union's internal market commissioner, Thierry Breton, stated that the crisis had revealed the need for Europe to boost its domestic production capacity in key sectors and to look “extremely carefully at the behaviour of every country where we have a supply chain” (quoted in Fleming and Peel 2020). The Japanese government has also assigned some of its COVID-19 stimulus money for assisting Japanese firms to make their supply chains more resilient and has announced extensive new restrictions on foreign investment in key sectors of the Japanese economy (Lewis 2020). Trump administration officials such as Peter Navarro have also argued that the crisis reinforces the need to reduce American dependence on global supply chains to “defend our citizens” (quoted in Politi, Williams, and Cookson 2020). The US trade representative Robert Lighthizer (2020) has similarly noted that American businesses have been “held hostage to decisions made by foreign governments about whether their suppliers are `essential' or not” and argued that “[t]he era of reflexive offshoring is over” and that it is time to “[b]ring the jobs back to America.”

It is important not to overstate the support for national self-sufficiency emerging from the COVID-19 crisis. In most cases, policymakers are interested in enhancing self-sufficiency in gradual and selective ways rather than advocating for the rapid introduction of a more ambitious autarkic vision. Like Oldenberg in the late nineteenth and early twentieth centuries, they recognize the enormous difficulties of trying to implement the latter in the context of today's deeply integrated global economy. The difficulties have been only compounded since Oldenberg's day by the contemporary globalization of finance and the emergence of dense and extensive global supply chains. Contemporary advocates of autarky, thus, find themselves in a much more challenging global environment than that of their predecessors.

It is not surprising, then, that even strong promoters of the idea of self-sufficiency, such as Modi (2020), conceptualize it in a very loose way. For example, after trumpeting the need for local economic activity, he highlighted how “[s]elf-reliance also prepares the country for a tough competition in the global supply chain.” But it is worth noting that prominent members of Modi's political party have also tied his endorsement of “self-sufficiency” to Gandhi's idea of *swadeshi* economics (Chari 2020). The echoes of past autarkic thought are also evident elsewhere in the renewed invocations of Keynes’ 1933 article (e.g., van Barneveld et al. 2020).

## Conclusion

The cover of *The Economist* magazine quoted at the start of this article was titled “Goodbye globalization: the dangerous lure of self-sufficiency.” The magazine summed up well the new political salience of the idea of national self-sufficiency in the contemporary age. As I have shown, however, ours is not the first era in which this idea has found support. There is a long lineage of thinkers dating back to the seventeenth century who have made a case for a high degree of national self-sufficiency. In many countries at different historical moments, the ideas of autarkists had at least as important a place in political debates as the other better-studied ideologies such as economic liberalism, neomercantilism, and Marxism. What precisely has been the intellectual “lure” of this idea?

The first and most common rationale has been a desire for insulation from foreign economic influences. Autarkists have been concerned about issues such as: vulnerabilities arising from unstable international markets, economic exploitation by foreigners, the impact of foreign competitive pressures on domestic firms and social groups, and especially external economic constraints on the ability of societies to pursue or maintain distinct domestic socioeconomic goals. Although thinkers have prioritized each of these concerns differently, the last one was most frequently cited by the thinkers analyzed in this article before the contemporary era. There was, however, enormous variety in the domestic socioeconomic goals that each of those thinkers hoped could be more easily pursued in an autarkic environment. These goals ranged from Kaempfer's centralized absolutism to Rousseau's decentralized republicanism, from Fichte's ambitious industrial planning to Müller's and Oldenberg's conservative agrarianism, from Aizawa's and Lee's Confucian visions to Kaiseki's ecological Buddhism, from Sekyi's conservative African developmentalism to Wang and Chen's Chinese anti-imperialism, from Gandhi's decentralist rural-centered vision to Aydemir's and Kim's authoritarian industrial developmentalism, and from Keynes’ managed liberal capitalism to Zimmermann's fascism.

The second rationale for national self-sufficiency has been the desire to insulate countries from foreign political and cultural influences that were associated with international economic relationships. Some autarkists have worried about political influences arising from countries’ economic dependence on foreign supplies and markets as well as from the possibility that economic exchanges might enable foreigners to promote domestic subversion or to lay a foundation for future annexation. Culturally, some thinkers have been concerned that foreign economic ties would undermine national identities and/or expose a country to undesirable foreign ideas and customs. Historically, the latter issue was raised particularly by non-Western thinkers who worried about the cultural implications of their country's participation in a Western-dominated world economy. Once again, however, thinkers have disagreed about the relative importance of these issues and many autarkist thinkers have not expressed these concerns at all. Indeed, some thinkers such as Fichte and Keynes strongly opposed the goal of cultural insulation, insisting that national economic self-sufficiency should not restrict the sharing of ideas and culture between countries.

Finally, some autarkists have seen national self-sufficiency as a force for international peace. For contemporary readers, this rationale may be the most surprising since the entire post-1945 international economic order has been premised on the opposite belief that economic openness brings peace. That belief stemmed from a liberal interpretation of the history of the 1930s, an interpretation that was strengthened by the endorsement of military expansionism by fascist autarkic thinkers in the interwar years. As we have seen, some earlier autarkic thinkers also supported war and/or military expansion, such as Fichte (although only in a short-term transition phase), Aiwaza, and Oldenberg. But there have also been many thinkers who associated autarky with international peace. Thinkers as diverse as Kaempfer, Fichte (over the longer term), Sekyi, Gandhi, and Keynes argued that this policy would diminish the reasons for conflict and war that arise from economic interdependence and commercial rivalry between countries. In addition, some have argued that autarkic societies were less likely to be attacked (Rousseau) and/or more able to defend themselves in the event of an attack (Kaempfer, Rousseau, Lee, Gandhi, Wang and Chen, Kim).

In outlining these various rationales for autarky, thinkers have not always have the same conception of national self-sufficiency. As just noted, disagreements have existed over whether autarky should include restrictions on the movement of ideas and culture. Some thinkers have also put more emphasis on the importance of self-sufficiency in specific sectors such as food (Rousseau, Oldenberg, Aizawa, and contemporary advocates of food sovereignty) or money and finance (Fichte, Müller, Paul, Keynes). More generally, differences have also existed between those with quite ambitious conceptions of total autarky and those who made more allowance for some enduring international economic flows and/or who promoted autarky more as a long-term goal than an immediate one. These various differences have related not just to the specific goals of various thinkers but also to the different temporal contexts in which they have been writing. The distinctiveness of different versions of autarkic thought has also been heavily influenced by specific country contexts. Indeed, as far back as Kaempfer, autarkic thinkers have recognized that some countries were better suited to implement autarkic policies than others.

The history of autarkic thought is, thus, a rich and diverse one that deserves more attention than it has received to date from scholars of IPE (as well as those interested in the history of economic thought and intellectual history). In addition to beginning to fill this gap in existing literature, this article has also sought to contribute to scholarship attempting to overcome the Western-centric nature of much IPE scholarship. Western-centrism is particularly pronounced in way that IPE scholars discuss the deep history of political economy. In most textbooks and courses, students are introduced to this history through a long line of Western thinkers such as Jean-Baptiste Colbert, Adam Smith, David Ricardo, Alexander Hamilton, Friedrich List, Karl Marx, Vladimir Lenin, Keynes and so on. This approach misses many key contributions made to the history of political economy by non-Western thinkers.

In the case of autarkic thought, these contributions were particularly important. As I have shown, some non-Western autarkists drew on Western thought, such as Shizuki's interest in Kaempfer's ideas, Francia's use of Rousseau, Paul's citing of Fichte's work, and the inspiration drawn from Italian fascism by Wang and Chen as well as Aydemir. But other non-Western thinkers developed innovative autarkic ideas without reference to Western ideas on the topic. Ideas also flowed in the other direction, as in the important case of Kaempfer who drew inspiration from his Japanese experience to become one of the key pioneers of European autarkic thought. The importance of embracing a more global approach to intellectual history, then, is not just that it highlights the contributions of non-Western thinkers but also that it helps us to recognize how “global conversations” about IPE issues have a deep history.^15^

Finally, this article also aims to provide some insights for growing IPE literature analyzing the politics of de-globalization in the contemporary age. Much of the emerging literature on this topic analyzes the changing interests, coalitions, institutions, market trends, and power structures that are encouraging de-globalization. However, as *The Economist* highlights, a key cause of contemporary de-globalization is also ideational: the growing attraction of the idea of national self-sufficiency. The history of autarkic thought provided in this article provides some historical context within which to understand this ideational phenomenon. Contemporary advocates of greater national self-sufficiency often highlight similar rationales for this policy as those raised in the past. Some also draw explicit inspiration from this history, even if the context for promoting autarkic policies is a more challenging one today. For this very practical reason, IPE scholars and students need to become more familiar with this neglected dimension of the intellectual history of political economy.

## Footnotes

^1^For a more detailed discussion of “neomercantilism,” see Helleiner (forthcoming). Although I use the phrase “national” self-sufficiency in the context of autarkic thought, it is important to note that some of the early thinkers I discuss did not have nationalist worldviews.

^2^Earlier thinkers, such as Antoine de Montchrétien in 1615, advocated autarky as a temporary policy before turning to more outward-oriented mercantilist policies (Palatano 2016).

^3^Kaempfer's original German language manuscript was finally published in the late 1770s (Bodart-Bailey 1999).

^4^During his time in Japan, Kaempfer had travelled beyond Nagasaki with the head of the Dutch mission on latter's annual visit to the shogun and spent several weeks in Edo (Goodman 2005, 364).

^5^For Rousseau's knowledge of Kaempfer's work, see, for example, Mervert (2009, 322, 328fn33).

^6^See especially the views of Confucian scholar and official Arai Hakuseki who tightened trade controls in 1709–1716 (e.g., Nakai 1988, 107–13).

^7^Shizuki's work was published just before Perry's arrival, while Aizawa was published in 1857.

^8^Francia was very dismissive of the quality of British textiles (which increasingly penetrated other Latin American markets at this time), calling them “rags” whose colours “fade upon the first washing” in contrast to “honest cloth” of Paraguayan looms (quoted on Robertson [1939]1970b, 227–28).

^9^The essay was modified slightly in each of these publications (e.g., Nolan 2013). I have quoted from the American version published in *The Yale Review*.

^10^This criticism was edited out when Keynes’ essay was reprinted in Germany (Borchardt 1990). In Keynes's initial version of the essay, he had referred to “the blond beasts of Germany” (quoted in Nolan 2013, 84).

^11^His essay was initially delivered as a lecture in Ireland in April 1933. It is often forgotten that the lecture included criticism of the goal of national self-sufficiency in the Irish context (Nolan 2013, 62–90).

^12^For the Nazi interest in Müller, see Szejnmann (2013, 362).

^13^Before the war, Stalin had promoted the idea of “socialism in one country” as a tactical response to the fact that the Russian revolution of 1917 had not been followed by similar revolutions in Europe and that socialist Russia was thus surrounded by hostile capitalist powers.

^14^For Trump's neomercantilist views, see Helleiner (2019) and Miller (2018).

^15^For the need for “global conservations” in contemporary IPE, see, for example, Tussie and Riggirozzi (2015) and Deciancio and Quilconi (2020).
